# Study on automatic lithology identification method while drilling based on acoustic pressure-rock physics parameters mapping

**DOI:** 10.1371/journal.pone.0330037

**Published:** 2025-11-07

**Authors:** Wei Jiang, Qingfeng Wang, Baoyong Yan, Yang Liu, Shuhan Shi, Hong Fu

**Affiliations:** 1 China Coal Research Institute, Beijing, China; 2 China Coal Technology and Engineering Group Chongqing Research Institute Co., Ltd., Chongqing, China; 3 School of Emergency Management and Safety Engineering, China University of Mining and Technology (Beijing), Beijing, China; Henan Polytechnic University, CHINA

## Abstract

The lithology identification while drilling is a critical component of intelligent coal mine exploration. Investigating automatic lithology identification methods is of great significance for enhancing reservoir prediction accuracy and the automation level of drilling exploration. This study proposes a novel method for automatic lithology identification while drilling based on the mapping relationship between acoustic pressure and rock physics parameters. First, core samples were collected from an operational mine borehole to prepare homogeneous (single lithology) and layered (composite lithology) rock specimens, providing reliable materials for drilling experiments. Second, a full-scale laboratory drilling system was designed and constructed, providing a robust dataset for time-frequency analysis with strong engineering applicability. Furthermore, a quantitative fitting model between acoustic pressure and rock physics parameters was constructed, and the physical mechanism between acoustic pressure and rock physics parameters was revealed. Finally, The mapping relationship between acoustic pressure and physics parameters was established, an automatic lithology identification algorithm was developed based on this mapping relationship. The results demonstrated that the acoustic pressure can be used as an effective response feature for identification of drilling lithology. The proposed method achieved recognition accuracies of 47%, 58%, 53%, 48%, 66%, and 71% for sandy mudstone, coal, mudstone, shale, limestone, and granite. The existence of the perforated transition zone does not affect the identification of lithology by the automatic identification algorithm. This research introduces a novel approach for lithology identification while drilling, which is pivotal for advancing the intelligent development of coal mine exploration.

## 1. Introduction

As a cornerstone of the global energy and industrial system, coal demand reached a record high of 8.77 billion tons in 2024, placing greater demands on its exploration and extraction [1]. With the progressive depletion of shallow coal resources, exploration is shifting towards unconventional areas with more complex geological conditions and deeper burial depths [2,3]. This shift not only significantly increases exploration risks and costs but can also lead to major safety accidents, severely constraining the safe and efficient extraction of coal resources. Therefore, developing advanced technologies for real-time, accurate, and objective identification of subsurface geological information is of great theoretical and practical significance for improving coal reservoir prediction, ensuring drilling safety, and achieving automated exploration.

Currently, primary methods for lithology identification in coal mines include traditional coring [4], borehole imaging [5], geophysical exploration [6], and methods based on the dynamic response of drilling parameters [7]. Traditional methods like core drilling provide direct rock samples but suffer from low efficiency, high labor intensity, high costs, and the inability to provide continuous formation information [8]. Borehole imaging techniques have significantly improved exploration speed and safety [9], and their rich image data offer more possibilities for analysis [10–12]. However, their imaging quality and reliability can be compromised by complex engineering conditions such as borehole collapse, drilling fluid contamination, and long-distance drilling. Geophysical methods (e.g., seismic, magnetic, and electrical exploration) are widely used for large-scale geological prospecting in coal mines and are effective for reservoir prediction [13,14] and formation identification [15]. Nevertheless, these methods typically provide macroscopic information and cannot achieve real-time lithology identification during drilling, limiting their application in unconventional coal areas with greater geological complexity.

Against the backdrop of rapid advancements in artificial intelligence, methods based on the dynamic response of drilling parameters have gained widespread attention in the fields of intelligent mining and smart drilling due to their continuous data acquisition, strong correlation with rock formations, and excellent anti-interference capabilities. Many scholars have utilized drilling parameters such as specific energy [16,17], torque and thrust [18], acoustic emissions [19], and vibrations [20,19] for lithology identification, with simulated drilling experiments validating their feasibility. However, current research using parameters like specific energy, torque, and thrust has several limitations:

(1) Existing drilling simulation experiments often use artificially synthesized rock samples [21,22], whose physical and chemical properties differ significantly from actual in-situ rock, failing to objectively replicate drilling conditions in complex geological settings.
(2) Most studies rely on small-scale drilling rigs [23], where the dimensions of the drill bit, rod, and rig do not match those used in field engineering, making it difficult to reflect the actual dynamic responses. Furthermore, due to the shallow drilling depths in these experiments, drilling fluid circulation systems are often omitted [21], thus neglecting the influence of drilling fluid on tool forces, cuttings transport, and parameter measurement.
(3) Current research predominantly focuses on the qualitative correlation between drilling parameters and lithology types. The quantitative relationship between lithology and key physics parameters—such as tensile strength, cohesion, and elastic modulus—remains underexplored. Moreover, existing studies often employ neural networks [24,25] or deep learning methods [26] for identification, which require extensive measured data for model training, leading to high data acquisition and processing costs.

Addressing these gaps, this study utilizes acoustic pressure generated during drilling as the primary response feature for lithology identification. We propose an automatic lithology identification method based on the mapping between acoustic pressure and rock physics parameters. First, core samples from an operational mine were used to prepare homogeneous and layered rock specimens, ensuring the reliability of the experimental materials. Second, a full-scale laboratory drilling system that closely mimics field conditions was designed and constructed to guarantee the engineering applicability of the experimental data. Furthermore, we established optimal fitting models to reveal the physical mechanisms linking acoustic pressure and rock physics parameters, providing a theoretical foundation for the identification algorithm. Finally, an algorithm that does not rely on large datasets was developed. This research aims to provide a novel method for intelligent coal exploration and to promote the development of automated and intelligent lithology identification technologies.

## 2. Methods

**Fig 1:**
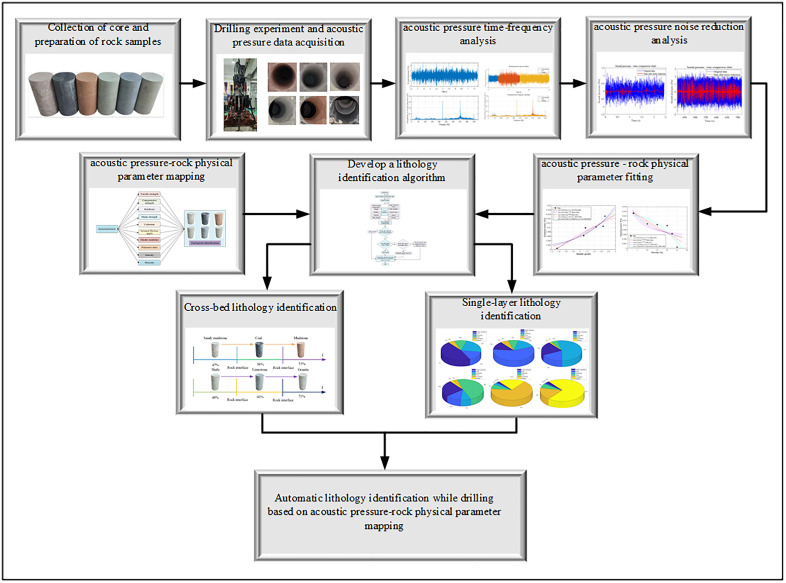
The workflow of the research method.

The methodology of this study encompasses experimentation, data analysis, and algorithm development, with the workflow illustrated in Fig 1. The main steps are as follows:

(1) Collect rock cores from an actual mine borehole to prepare homogeneous (single-lithology) and layered (composite) specimens, providing reliable materials for drilling experiments.
(2) Design and construct a full-scale drilling experimental system. Conduct drilling tests on both homogeneous and layered specimens to acquire acoustic pressure data during drilling, which serves as the basis for subsequent analysis.
(3) Perform time-domain and frequency-domain analyses on the acoustic pressure data from both drilling scenarios to validate its suitability as a response feature for lithology identification.
(4) Apply a denoising process to the raw acoustic pressure data to remove noise, providing a clean signal for building the fitting models between acoustic pressure and rock physics parameters.
(5) Develop fitting models between acoustic pressure and rock physics parameters to analyze their mapping relationship, providing a theoretical foundation for the identification algorithm.
(6) Develop an automatic lithology identification algorithm based on the established mapping relationship. Analyze the identification results for both homogeneous and layered drilling to validate the algorithm’s feasibility and assess the impact of transition zones in layered formations.

### 2.1 Experimental methods

#### 2.1.1 Experimental materials.

To ensure the reliability of the rock specimens, all raw core materials were sourced from the Zhangji drilling mine in Anhui Province, China. The materials included six rock types: sandy mudstone, coal, sandstone, shale, limestone, and granite. The composition of these materials is detailed in Table 1. The raw cores were processed by the Huashun Stone Factory in Chongqing, China, into homogeneous (single-lithology) and composite (three-lithology combination) specimens with dimensions of 300x300x600 mm, the size of each lithology sample in the composite lithology samples is 300x300x200 mm. The overall sealing method for composite lithology rock samples is as follows:

**Table 1:** Composition of core raw materials.

Core Type	Main mineral composition	Chemical composition
Sandy Mudstone	Clay minerals(50%−70%)、sand particles(30%−50%).	Main components: SiO_2_, Al₂O₃. Minor components: Fe_2_O_3_, CaO, MgO.
Coal	Clay minerals, quartz, carbonates, sulfides.	Organic components: C(50%−95%), H, O, N, S. Inorganic components: Si, Al, Ca, Fe, Mg.
Mudstone	Clay minerals(more than 50%), containing a small amount of fine quartz, feldspar.	Main components: SiO_2_, Al_2_O_3_. Minor components: Fe, Ca, Mg.
Shale	Clay minerals (40%−80%), quartz, feldspar.	Main components: SiO_2_, Al_2_O_3_. Minor components: Ca, Mg, Fe.
Limestone	Calcite (more than 50%), containing a small amount of dolomite, clay minerals, quartz.	Main components: CaCO_3_. Minor components: MgCO_3_, SiO_2_, Al_2_O_3._
Granite	Quartz(10%−50%)、potassium feldspar(30%−60%)、plagioclase(10%−30%),dark minerals(5%−15%).	Main components: SiO_2_(65%−75%)、Al_2_O_3_. Minor components: Fe、Mg.

**Fig 2:**
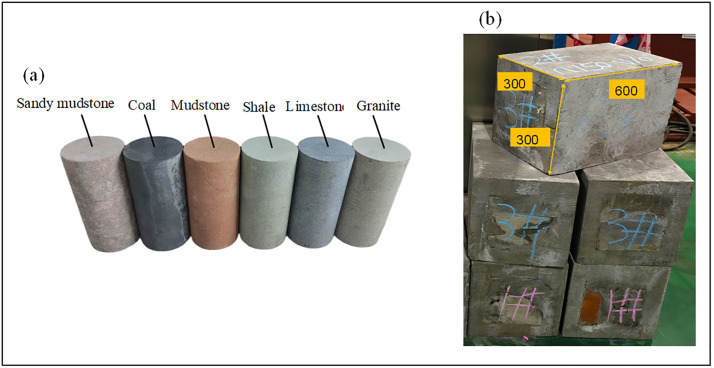
Rock sample model and rock sample finished product. (a) Sample model. (b) Sample product.

(1) Use a cutting machine to cut small samples from the entire core raw material, and process the small samples into single – lithology rock samples (300x300x200mm).
(2) Make a wooden trough pouring mold (300x300x600mm) with wooden boards, place the single – lithology rock samples into the mold in an orderly manner according to the experimental plan, and pour the grout prepared from CGM standard – type high – strength non – shrinkage grout until it covers the upper edge of the mold.
(3) Wait for the grout to initially set (for 48 hours), demold in a cool place and cure in ventilation for 10 days, so as to form a pouring layer with good integrity and realize the overall sealing of the composite rock sample. This pouring layer can ensure the consistency and continuity of drilling, and also provide confining pressure for the composite sample to simulate the real geological stress conditions. The models and finished specimens are shown in Fig 2.

#### 2.1.2 Measurement of rock physics parameters.

The key physics parameters of the rock specimens were measured, including tensile strength, compressive strength, hardness, shear strength, cohesion, internal friction angle, elastic modulus, Poisson’s ratio, density, and porosity. Tensile strength was determined using a direct tensile test. Compressive strength was obtained via a uniaxial compressive strength (UCS) test on standard samples. Hardness was measured using the scratch hardness method. Shear strength, cohesion, and internal friction angle were determined from triaxial compression tests. Elastic modulus and Poisson’s ratio were acquired from uniaxial compression tests with strain measurement. Density was calculated based on volume measurements. Porosity was determined by measuring water absorption to calculate pore volume. The measured physics parameters are presented in Table 2.

**Table 2:** Measured values of physics parameters of rock samples. (Tensile strength: TS. Compressive strength: CS. Mohs hardness: HM. Shear strength: τ, Cohesion: c. Internal friction angle: φ. Elastic modulus: E. Poisson’s ratio: ν. Density: ρ. Porosity: κ).

Rock type	TS (MPa)	CS (MPa)	HM	τ (MPa)	c (MPa)	φ (°)	E (GPa)	ν	ρ (g/cm³)	κ (%)
Sandy mudstone	2.4	22.5	2.7	11.5	3.9	20	6	0.3	2.15	20
Coal	0.56	6.8	1.5	3	1.2	15	2.72	0.35	1.35	25
Mudstone	2.1	20.2	2.5	8	2.8	16	4.5	0.3	2.1	22.5
Shale	4	25.5	3	14	5.5	24	12.5	0.33	2.45	15
Limestone	10	88	3.5	35	20	32.5	35	0.25	2.65	10
Granite	17.5	125	6.5	75	55	37.5	55	0.225	2.7	3

#### 2.1.3 Drilling tools.

**Fig 3:**
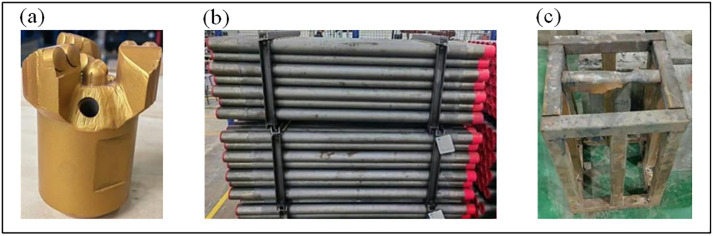
Drilling tools. (a) Three-wing diamond composite drill bit. (b) Standard drill rod. (c) specimen holder.

The drilling tools used in the experiment, including the drill bit, drill rod, and a specimen holder, were provided by the China Coal Technology and Engineering Group Chongqing Research Institute. To simulate field conditions as closely as possible, all tools were of standard engineering scale. A 94 mm diameter three-wing Polycrystalline Diamond Compact (PDC) bit (The manufacturing material is cemented carbide) was used to break the rock through rotational shearing and crushing. A 73 mm diameter standard drill rod (The manufacturing material is high-strength alloy steel) transmitted power and torque from the rig to the bit. A custom-fabricated specimen holder with internal dimensions of 300x300x600 mm and external dimensions of 400x400x700 mm was used to secure the rock specimen during drilling. The drilling tools are depicted in Fig 3.

#### 2.1.4 Drilling equipment.

The experiment was conducted using a ZYWL-4500 high-position, crawler-type, fully hydraulic drilling rig. The rig features a compact, integrated design. Its drilling inclination is adjusted by hydraulic cylinders, and a hydraulic turntable at its base allows for 360° rotation, enabling drilling in any direction. The specific operating conditions, measurement uncertainties, and calibration procedures of the drilling equipment are as follows:

(1) Operational Conditions: The working pressure of the hydraulic pump is controlled within the range of 16–20 MPa, and meanwhile, the flow rate of the hydraulic oil is maintained at 80–100 L/min, ensuring that the hydraulic system provides sufficient and stable power support for the drilling rig’s operations such as drilling and inclination angle adjustment.
(2) Measurement Uncertainty: A high-precision inclination sensor is installed on the drill pipe near the power head of the drilling rig. It has a measurement accuracy of ±0.1° and a repeatability error controlled within 0.05°, which fully ensures the accuracy of inclination measurement. The drilling depth is measured by a depth encoder, which has a resolution of 1 mm and a relative uncertainty of less than 0.2%, meeting the requirements for drilling depth measurement accuracy in the experiment.
(3) Calibration Protocol: The drilling speed of the drill bit is calibrated by the extension and retraction length of the oil cylinder. When the oil cylinder is advanced, the drill bit penetrates the rock sample. The drilling inclination angle is calibrated via the center of the rock sample. Before drilling, the drill bit is aligned with the center of the rock sample to complete the pre-drilling calibration.

**Fig 4:**
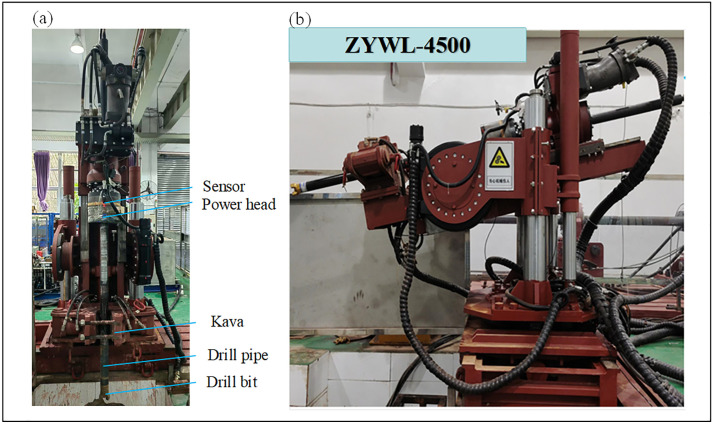
Drilling rig structure. (a) Main view of the drilling rig. (b) Side view of the drilling rig.

The structure of the rig is shown in Fig 4, and its main technical parameters are listed in Table 3.

**Table 3:** Main technical parameters of the drilling rig.

Technical parameters	Unit	Value
Maximum drilling depth	m	400
Opening diameter	mm	94
Final hole diameter	mm	94
Drill rod diameter	mm	73
Drilling inclination angle	^0^	−90−90
Drilling azimuth	^0^	0-360
Minimum opening height	mm	1250
Range of elevation	mm	1000
Rated output rotational speed	r/min	60-220
Rated output torque	N.m	1200-4500
Propulsion force	kN	110
Lifting force	kN	150
Normal speed	m/min	0-1.5
Feeding process	mm	650
Mainframe transportation dimensions	mm	3400x1000x1480
Mainframe external dimensions	mm	4127x1000x1850
Drilling rig power	kW	55
Overall weight of the machine	kg	6500

#### 2.1.5 Drilling system.

**Fig 5:**
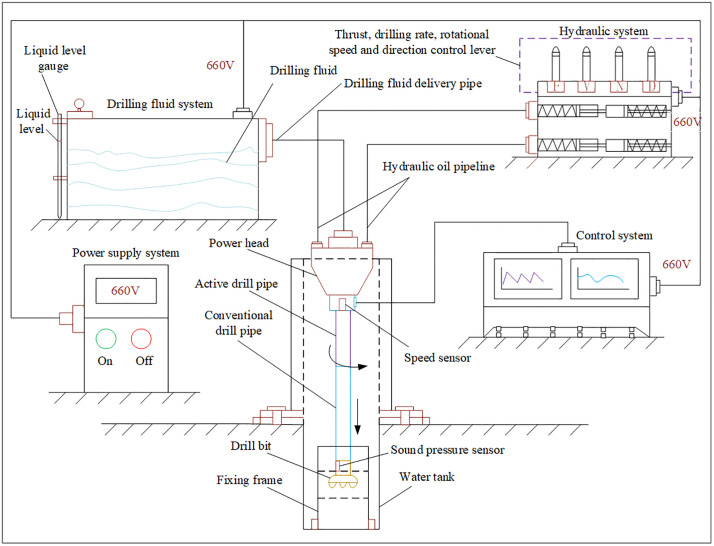
Composition of the drilling system.

A comprehensive drilling system was designed and constructed for the experiments, as shown in Fig 5. The system consists of five main subsystems: the Drilling Structure, Power Supply, Drilling Fluid, Hydraulic, and Control Systems. The Drilling Structure system is the executive part, comprising the power head, speed sensor, active drill rod, standard drill rod, acoustic pressure sensor, and drill bit. The Power Supply System controls the start and stop functions. The Drilling Fluid System, consisting of drilling fluid, a level gauge, and a fluid tank, cools the drill bit and transports cuttings out of the borehole. The Hydraulic System is the power source, providing drilling force and allowing adjustment of drilling parameters via control handles for thrust, rotation speed, and direction. The Control System monitors drilling parameters in real-time.

#### 2.1.6 Drilling experiments.

**Fig 6:**
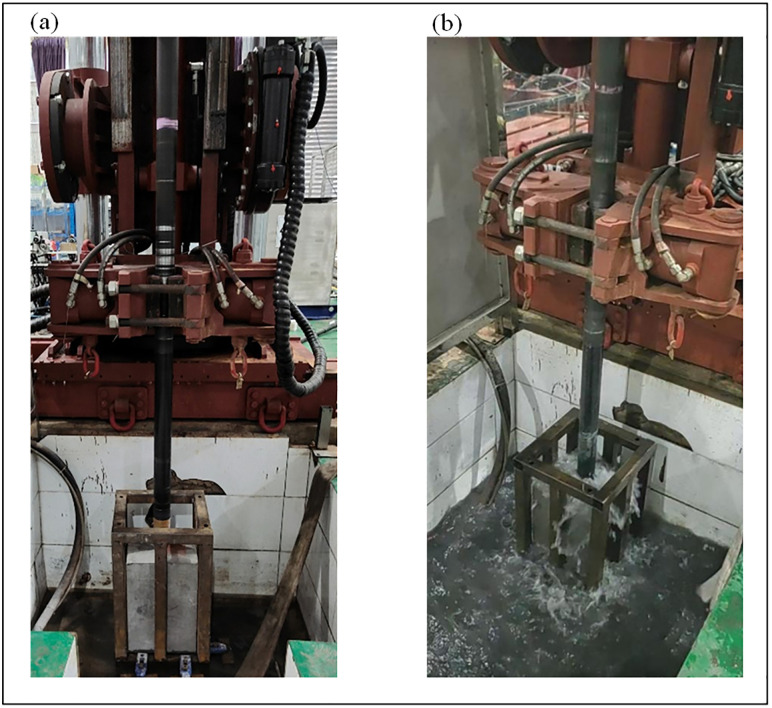
Drilling experiment. (a) Drilling positioning. (b) Rotary cutting drilling.

**Fig 7:**
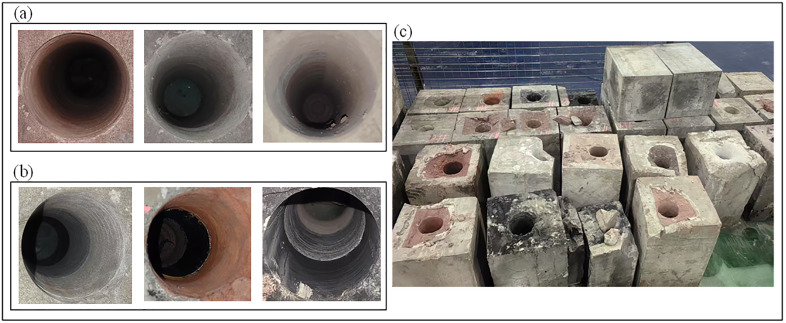
Drilling experiment results. (a) Single-layer drilling results of rock samples. (b) Through-layer drilling results of rock samples. (c) Completed rock samples.

The experiments were performed at the “Safety Access Analysis and Verification Laboratory for Coal Mine Gas Drainage and Coal Seam Permeability Enhancement Equipment.” The tests included both homogeneous drilling (in a single rock type) and layered drilling (through three combined rock types). The experimental setup and results are shown in Figs 6 and 7, respectively. The procedure was as follows:

(1) The experimental platform was assembled as shown in Fig 5, including all five subsystems and the installation of acoustic pressure and speed sensors.
(2) The power was activated, and the initial drilling inclination, azimuth, and hydraulic pressure were set.
(3) As shown in Fig 6(a), a rock specimen was placed in the holder. The holder was then lowered into a water tank using a crane, secured with bolts, and positioned for drilling.
(4) The drilling fluid system was activated to supply fluid through the bit nozzles. The power head was started, rotating the drill string. The hydraulic controls were used to set the rotation speed to 150 r/min, the penetration rate to 10 mm/s, and the thrust to 60 kN. Drilling commenced as shown in Fig 6(b).
(5) When adding or removing drill rods, the slips were used to secure the drill string before performing the connection/disconnection.
(6) After drilling through a specimen, it was replaced, and steps (3)–(5) were repeated until all experiments were completed.

### 2.2 Data analysis methods

#### 2.2.1 Acoustic pressure time-domain analysis.

This study generated over one million acoustic pressure data points. To intuitively understand the dynamic characteristics of the acoustic pressure signal over time, a time-domain analysis was necessary. This analysis helps to understand the propagation patterns of the acoustic pressure field during drilling in different rock types and directly observe the acoustic pressure intensity, providing a preliminary basis for assessing its viability as a lithology identification feature.

#### 2.2.2 Acoustic pressure frequency-domain analysis.

While time-domain analysis reveals temporal fluctuations, it can suffer from large signals masking smaller ones, thus failing to capture the detailed distribution of the signal’s components. Frequency-domain analysis, however, can identify weak yet significant signal components, providing a more comprehensive understanding. Therefore, a frequency-domain analysis was performed to further validate acoustic pressure as a response feature. A continuous-time signal can be transformed into the frequency domain using the Fourier Transform (FT) [27–29], as described by Equations (1) and (2).


$$x(t)=\int_{-\infty }^{+\infty }X(f)e^{j2\pi ft}df$$ (1)

$$X(f)=\int_{-\infty }^{+\infty }x(t)e^{-j2\pi ft}dt$$ (2)

Where *t* is the time-domain signal of acoustic pressure, *X(f)* is the frequency-domain signal of acoustic pressure, *X(f) is the Fourier transform of x(t)*, *x(t)* is the inverse Fourier transform of *X(f)*.

However, in practical applications, the acoustic pressure was sampled at discrete time intervals τ, therefore, the time-domain signal is transformed into the frequency domain using the Discrete Fourier Transform (DFT) [30,31], as shown in Equation (3).


$$X_{k}=\sum_{n=0}^{N-1}x_{n}e^{-j\frac{2\pi }{N}kn}(0\leq k<N)$$ (3)

Where *X*_*k*_ is the frequency-domain signal of acoustic pressure, *x*_*n*_ is the time-domain signal of acoustic pressure, *N* represents the acoustic pressure data volume, *k* represents the frequency index.

#### 2.2.3 Acoustic pressure signal denoising.

To achieve quantitative lithology identification in this study, it is essential to build accurate fitting models between acoustic pressure and rock physics parameters. The raw data collected by the sensor contained noise. To improve the accuracy of the fitting models, denoising was a necessary preprocessing step.

**Fig 8:**
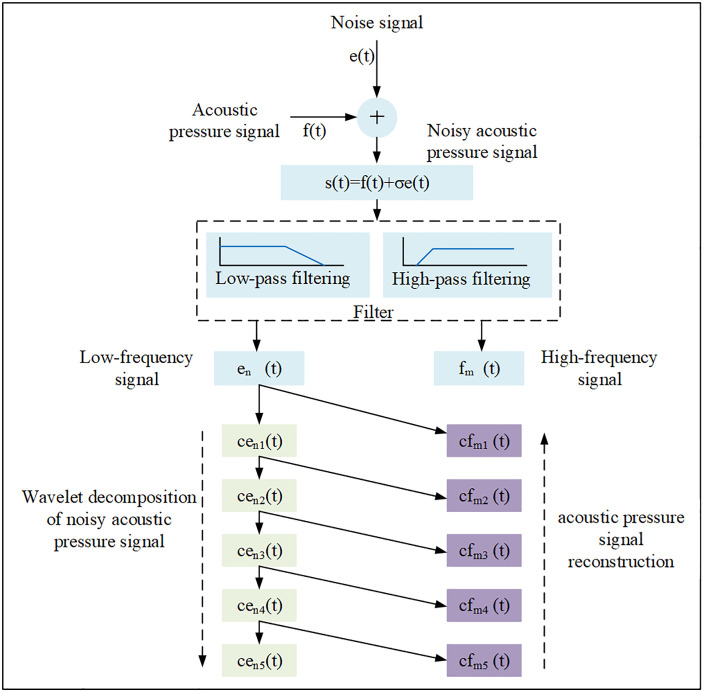
Wavelet decomposition and reconstruction methods for noisy acoustic pressure signals.

Considering the demands of practical applications, the denoising process needed to effectively remove noise while preserving the original signal’s integrity to ensure the broad applicability of the resulting models. Given the random and non-stationary nature of the noise, the Wavelet Transform (WT) was chosen for denoising [32,33]. The wavelet decomposition and reconstruction process is illustrated in Fig 8.

The specific steps were as follows:

(1) Signal Input: The noisy acoustic pressure signal is input.
(2) Initial Decomposition: Low-pass filtering extracts the low-frequency components e_n_(t),while high-pass filtering extracts the high-frequency components f_m_(t).
(3) Multi-level Decomposition: The signal is decomposed into multiple levels (set to 5 in this study), yielding detail coefficients(cf_m1_(t)、cf_m2_(t)、cf_m3_(t)、cf_m4_(t) and cf_m5_(t)) and approximation coefficients(ce_n1_(t)、ce_n2_(t)、ce_n3_(t)、ce_n4_(t) and ce_n5_(t)). The Daubechies 4 (db4) wavelet was used as the mother wavelet.
(4) Signal Denoising: According to the characteristics of the noisy acoustic pressure signal, the wavelet decomposition order is set to 5 levels, and the Daubechies 4 wavelet is adopted as the wavelet basis function.
(5) Signal Reconstruction: The denoised signal is reconstructed from the processed coefficients using the Inverse Wavelet Transform (IWT) [34].

#### 2.2.4 Construction of acoustic pressure-rock physics parameters fitting models.

Building fitting models between acoustic pressure and rock physics parameters is analogous to establishing quantitative relationships between well-logging data and formation properties. These models reveal the underlying physical mechanisms and are crucial for automating lithology identification.

In this study, the mean of the denoised acoustic pressure was taken as the characteristic response value for each rock type. The least-squares method was used to fit quantitative models between the mean acoustic pressure and ten physics parameters: tensile strength, compressive strength, hardness, shear strength, cohesion, internal friction angle, elastic modulus, Poisson’s ratio, density, and porosity. The process involved:

(1) Selecting a suitable fitting function based on the data distribution.
(2) Constructing an error function, specifically the sum of squared errors (SSE), as shown in Equation (4).

$$SSE=\sum {\left(y_{i}-{y_{a}}_{i}\right)}^{2}$$ (4)

Where y_i_ is the i-th observed value, y_ai_ is the i-th fitted value.

(3) Solving for the optimal parameters using analytical solutions (for linear fits) or iterative algorithms (for non-linear fits).
(4) Evaluating the goodness-of-fit using the coefficient of determination (R²), as calculated by Equation (5).

$$R^{2}=1-\frac{\sum {\left(y_{i}-y_{ai}\right)}^{2}}{\sum {\left(y_{i}-y_{b}\right)}^{2}}$$ (5)

Where R^2^ is the coefficient of determination, y_b_ is the average value.

### 2.3 Development of the automatic lithology identification algorithm

#### 2.3.1 Algorithm design.

The preceding analysis, based on time- and frequency-domain characteristics, established that acoustic pressure can serve as an effective response feature for lithology identification while drilling. Building on this premise, the noisy acoustic pressure signal was denoised, and fitting models between acoustic pressure and rock physics parameters were constructed. However, the methodology for leveraging these fitting models to achieve automatic lithology identification remains to be defined. Therefore, it is necessary to develop an algorithm that utilizes the established mapping for this purpose.

**Fig 9:**
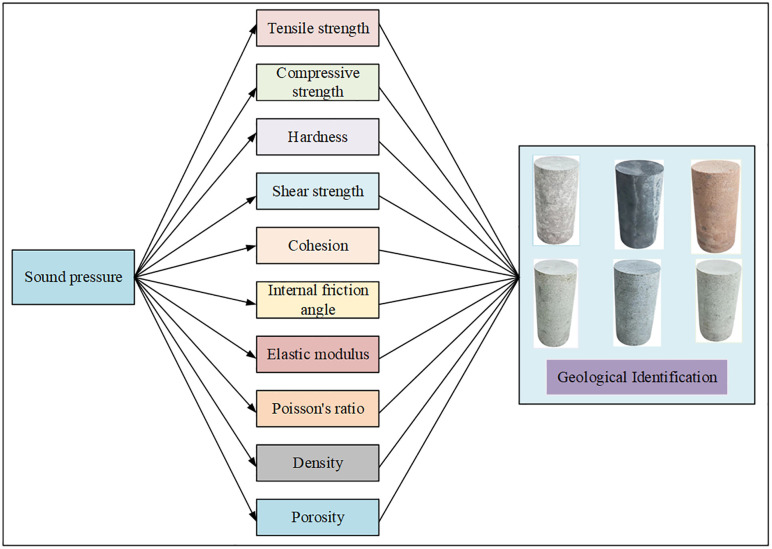
Mapping relationship between acoustic pressure and rock physics parameters.

In the study, we developed an algorithm based on the mapping relationship between acoustic pressure and rock physics parameters. As shown in Fig 9, based on the sound pressure-rock physical parameter fitting model, the physical parameters of borehole rock samples (tensile strength, compressive strength, hardness, shear strength, cohesion, internal friction angle, elastic modulus, Poisson’s ratio, density, porosity) can be correlated according to the sound pressure of the borehole rock samples, thereby realizing borehole rock sample identification.

**Fig 10:**
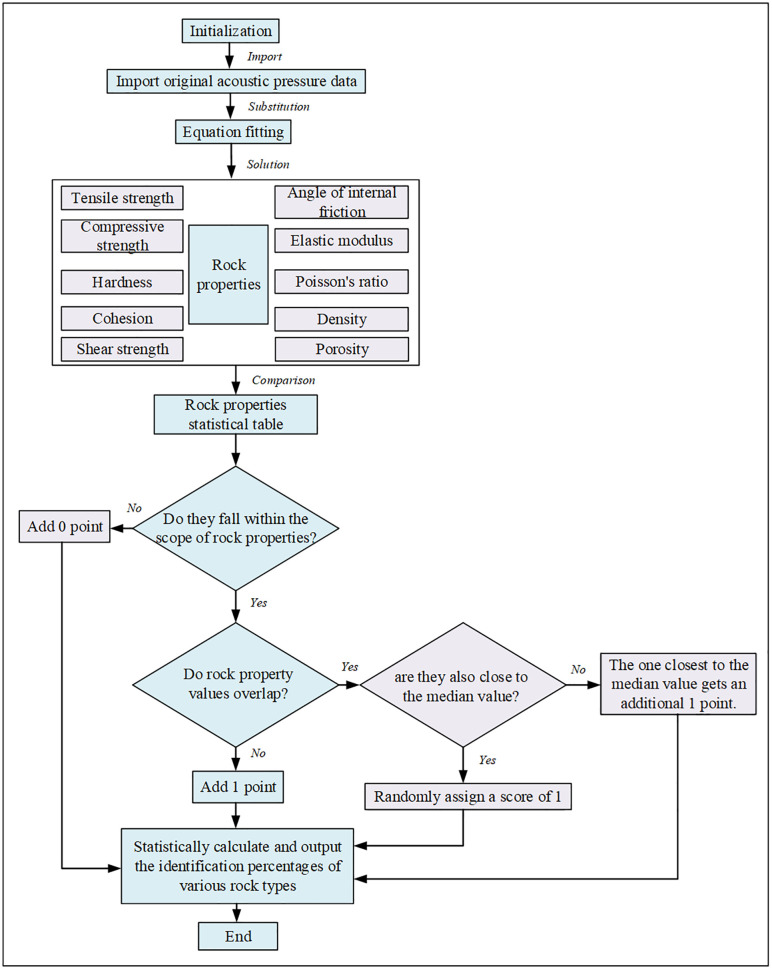
Automatic lithology recognition algorithm while drilling.

As shown in Fig 10, the algorithm operates as follows:

(1) Data Acquisition: Collect acoustic pressure data during drilling.
(2) Parameter Calculation: Calculate the moving average of the acoustic pressure data (e.g., one average value per 50 data points). Input this average value into the established fitting models to compute the corresponding values for the ten rock physics parameters.
(3) Parameter Comparison: Compare the calculated parameter values with a statistical database of known rock properties (Table 4) [35–37].
(4) Scoring and Identification: For each calculated parameter, a score is assigned. If a calculated value falls outside the range of any rock type in the database, it scores 0. If it falls within the range of a single rock type, that rock type scores 1 point. If it falls within the overlapping ranges of multiple rock types, the point is awarded to the rock type whose range median is closest to the calculated value. If the proximity is equal, the point is randomly assigned among the tied rock types.
(5) Tallying: The scores for each rock type are tallied over a given drilling interval. The rock type with the highest percentage of total points is identified as the lithology.
(6) Iteration: Steps (2)–(5) are repeated continuously as drilling progresses.

**Table 4:** Statistical table of petrophysics parameters.

Rock type	TS (MPa)	CS (MPa)	HM	τ (MPa)	c (MPa)	φ (°)	E (GPa)	ν	ρ (g/cm³)	κ (%)
Sandy mudstone	1-5	10-30	2-3	5-15	2-5	15-25	2-10	0.25-0.35	2.0-2.3	15-25
Coal	0.5-3	5-20	1-2	2-8	1-3	10-20	0.5-5	0.30-0.40	1.2-1.5	20-30
Mudstone	1-4	8-25	2-3	4-12	1-4	12-20	1-8	0.28-0.38	2.0-2.2	15-30
Shale	2-6	15-40	2-4	8-20	3-8	18-30	5-20	0.25-0.35	2.3-2.6	10-20
Limestone	5-15	50-150	3-4	20-50	10-30	25-40	20-50	0.20-0.30	2.5-2.8	5-15
Granite	10-25	100-250	6-7	50-100	30-80	30-45	40-70	0.20-0.25	2.6-2.8	1-5

#### 2.3.2 Analysis of inter-layer drilling identification.

**Fig 11:**
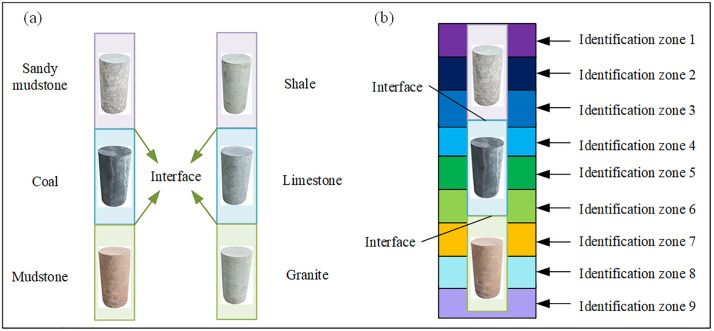
Inter-layer drilling identification. (a) Inter-layer drilling transition zone (b) Inter-layer drilling identification area.

During layered drilling, the bit transitions from one rock type to another (Fig 11(a)). Due to the different physics properties, the acoustic pressure signal changes at the transition zone. Consequently, the physics parameters calculated from the acoustic pressure in this zone may match the previous rock type, match the subsequent rock type, or match neither. As shown in Fig 11(b), although these ambiguous identifications occur at the transition zone (e.g., recognition zones 3 and 6), they are transient events. Since the final identification is based on the cumulative score percentage over the entire section, these isolated instances at the boundaries do not significantly impact the overall identification accuracy for each layer.

## 3. Results and analysis

### 3.1 Time and frequency domain features

#### 3.1.1 Features of homogeneous drilling.

**Fig 12:**
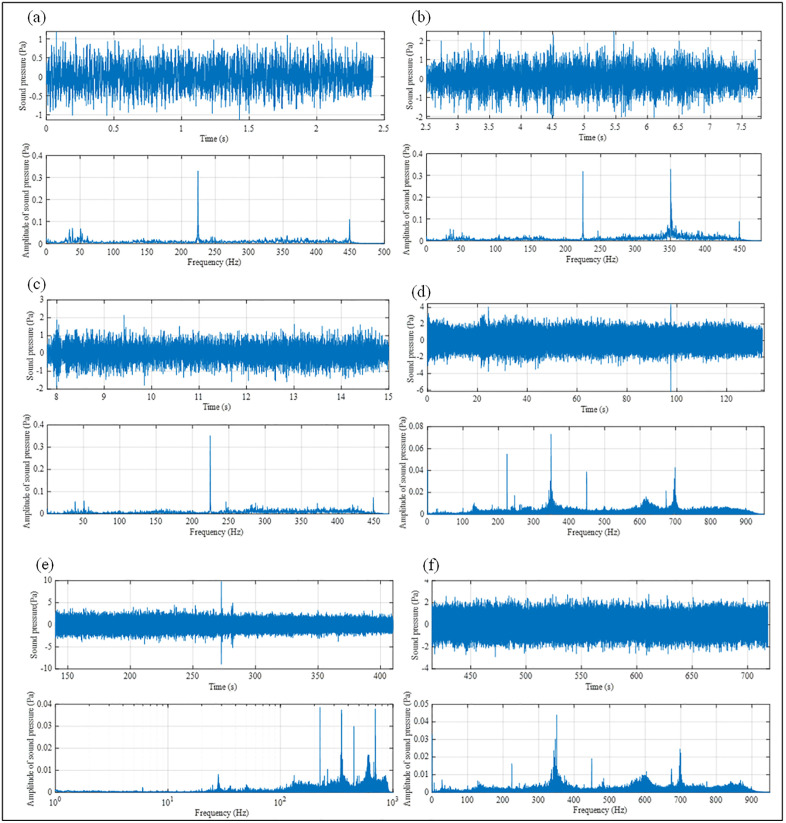
Time- and frequency-domain features of homogeneous drilling. (a) Sandy mudstone. (b) Coal. (c) Mudstone. (d) Shale. (e) Limestone. (f) Granite.

The time-domain acoustic pressure signals for the six rock types are shown in Fig 12. Rock types with greater differences in properties (e.g., coal vs. granite) exhibited more distinct differences in acoustic pressure intensity. Conversely, rocks with similar properties (e.g., shale vs. granite in this representation) showed less obvious differences. The occurrence of this phenomenon can be explained by the theory of wave propagation in inhomogeneous media. When drilling into rock samples with significant differences in properties, the impedance mismatch at the rock interface causes elastic waves to undergo significant reflection and refraction, resulting in obvious fluctuations in sound pressure intensity in the time domain. In contrast, for rock samples with small property differences, the change in interface impedance is minor, leading to little reflection and refraction of elastic wave energy, and thus the sound pressure intensity does not change significantly. Therefore, for rock samples with large property differences, lithology can be preliminarily distinguished through the time-domain characteristics of sound pressure. However, for rock samples with small property differences, it is difficult to directly determine lithology solely by relying on the time-domain characteristics of sound pressure, and frequency-domain analysis needs to be combined.

Based on the sound pressure frequency-domain characteristics of the 6 rock samples in Fig 12, all the different rock samples exhibit consistent sound pressure peaks at 225 Hz and 450 Hz. Combined with the mechanical system vibration theory, these frequency bands are the natural resonance frequencies of the drilling system, which are determined by the inherent properties (such as mass and stiffness) of components like the drilling rig and drill pipe, and are independent of lithology. In addition, different rock samples have distinct sound pressure spectrum characteristics. At 350 Hz, the sound pressure peaks of different rock samples show significant differences. According to the rock physics and acoustic wave propagation theory, the physical properties of rocks (such as elastic modulus and density) affect the propagation and attenuation of acoustic waves in them, which in turn leads to different sound pressure peaks of different rock samples at specific frequencies (e.g., 350 Hz). This indicates that the sound pressure peak in the frequency domain at this frequency can be used as an indicator for lithology identification. The sound pressure peaks of the 6 different rock samples are shown in Table 5.

**Table 5:** Peak acoustic pressure of six different rocks.

Rock type	Frequency (Hz)	Peak acoustic pressure (Pa)
Sandy mudstone	350	0.0076
Coal	350	0.3288
Mudstone	350	0.0069
Shale	350	0.0731
Limestone	350	0.0375
Granite	350	0.0439

#### 3.1.2 The time and frequency domain features of layered drilling.

**Fig 13:**
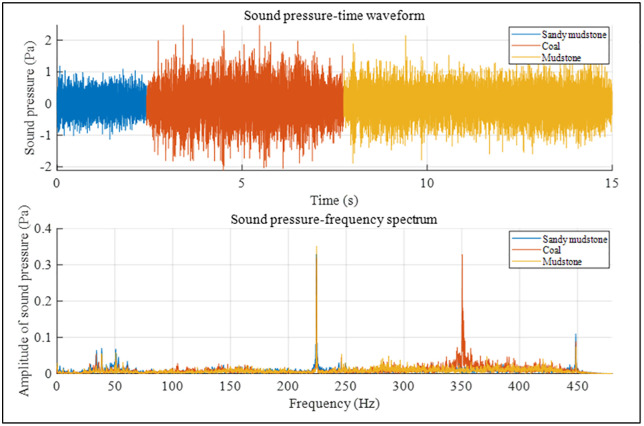
Sequence of layered drilling: sandy mudstone-coal-mudstone.

**Fig 14:**
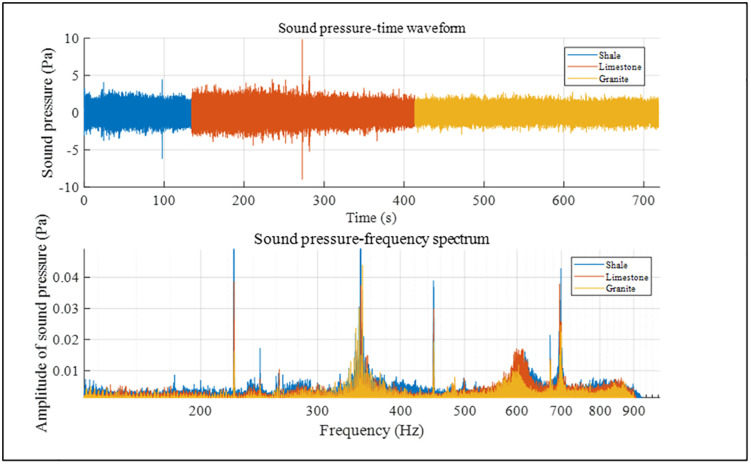
Sequence of layered drilling: shale-limestone-granite.

In the previous text, we separately analyzed the time-domain and frequency-domain characteristics of acoustic pressure in six different rock samples and initially determined that acoustic pressure can be used as a response feature for lithology identification while drilling. To intuitively compare the sound pressure time-domain and frequency-domain characteristics of different rock samples, we analyzed the sound pressure time-domain and frequency-domain characteristics of cross-layer boreholes, as shown in Figs 13 and 14. The sound pressure time-domain waveform shows obvious segmentation corresponding to rock layers, with significant differences in amplitude and fluctuation characteristics due to different lithologies (e.g., the waveform in coal and granite sections has large amplitude and intense fluctuation, while the opposite is true for the shale section). The peak frequency and amplitude distribution of the frequency-domain spectrum also change with rock layers. The occurrence of this phenomenon is consistent with the theories of wave propagation in layered media and acoustic resonance. Different lithologies (such as elastic modulus and density) lead to impedance mismatch between layers, causing reflection and transmission of elastic waves and thus changes in time-domain waveforms. Meanwhile, in the acoustic system composed of multi-layered rocks, each layer acts as a resonator with a different natural frequency and interacts with one another, resulting in differences in frequency-domain characteristics. Based on the above analysis, the time-domain and frequency-domain characteristics of different rock samples are significantly different, which further indicates that sound pressure can be used as a response characteristic for lithology identification while drilling.

### 3.2 Acoustic pressure denoising analysis

**Fig 15:**
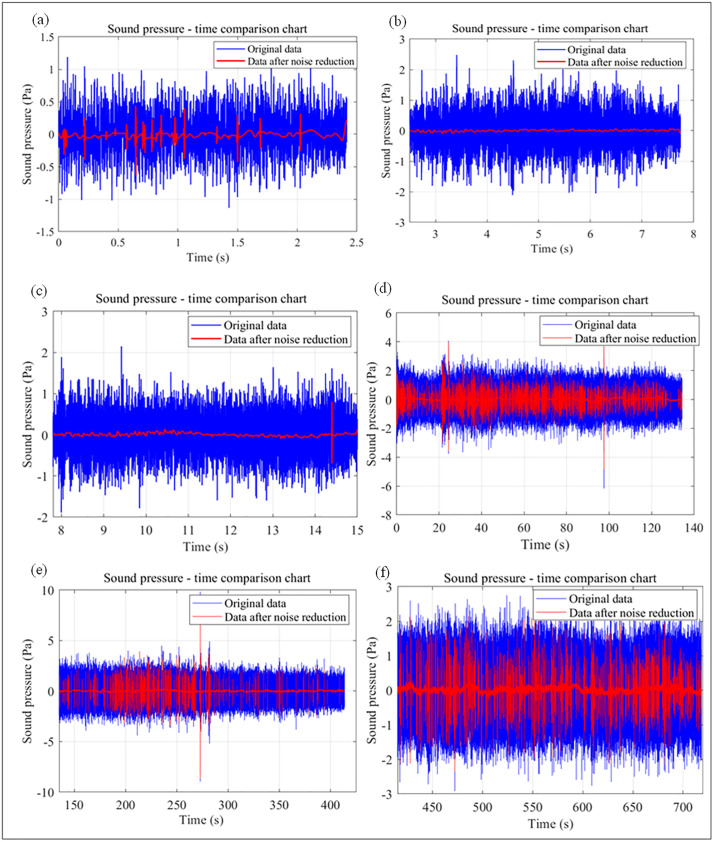
Comparison of time-domain features of acoustic pressure before and after denoising of different rock samples. (a) Sandy mudstone. (b) Coal. (c) Mudstone. (d) Shale. (e) Limestone. (f) Granite.

Wavelet transform was used for denoising the noisy sound pressure data. Wavelet transform can decompose a signal into different frequency bands; by setting a threshold, it can suppress or eliminate the coefficients corresponding to high-frequency noise while retaining the coefficients corresponding to low-frequency useful signals. In Fig 15, the original sound pressure data (blue curve) exhibits severe high-frequency fluctuations. After denoising (red curve), the high-frequency fluctuations are significantly reduced, and the irregular fluctuations caused by noise are effectively suppressed. This indicates that the denoising process has effectively removed high-frequency noise, providing more accurate data support for the construction of the sound pressure-rock physical parameter fitting model. The average values of sound pressure data of the 6 different rock samples before and after denoising are shown in Table 6.

**Table 6:** Average value of acoustic pressure data before and after denoising.

Rock type	Average value of original acoustic pressure data (Pa)	Average value of denoised acoustic pressure data (Pa)
Sandy mudstone	0.009822897	0.00984697
Coal	0.00053237	0.000533671
Mudstone	0.00621654	0.006338573
Shale	0.010232557	0.010235679
Limestone	0.010594841	0.010590083
Granite	0.015159605	0.015159769

According to the average values of sound pressure data before and after denoising in Table 6, there are slight differences between the average sound pressure data after denoising and the original data. For example, the original average value of sandy mudstone is 0.009822897 Pa, and it becomes 0.00984697 Pa after denoising; the original average value of coal is 0.00053237 Pa, and it becomes 0.000533671 Pa after denoising. These slight differences reflect that wavelet transform has minimal impact on the overall level of useful signals while removing noise, which is consistent with the optimal approximation theory of wavelet transform.

### 3.3 Analysis of acoustic pressure-physical parameter fitting

The preceding analysis validated acoustic pressure as an effective response feature for lithology identification. Furthermore, after applying a wavelet transform-based method to denoise the raw signal, a clean acoustic pressure dataset was obtained. Building upon this foundation, the subsequent step involves constructing fitting models between acoustic pressure and rock physics parameters. These models are intended to reveal the underlying physical mechanisms and to provide a theoretical basis for developing an automatic identification algorithm based on the acoustic pressure-physical parameter mapping.

**Fig 16:**
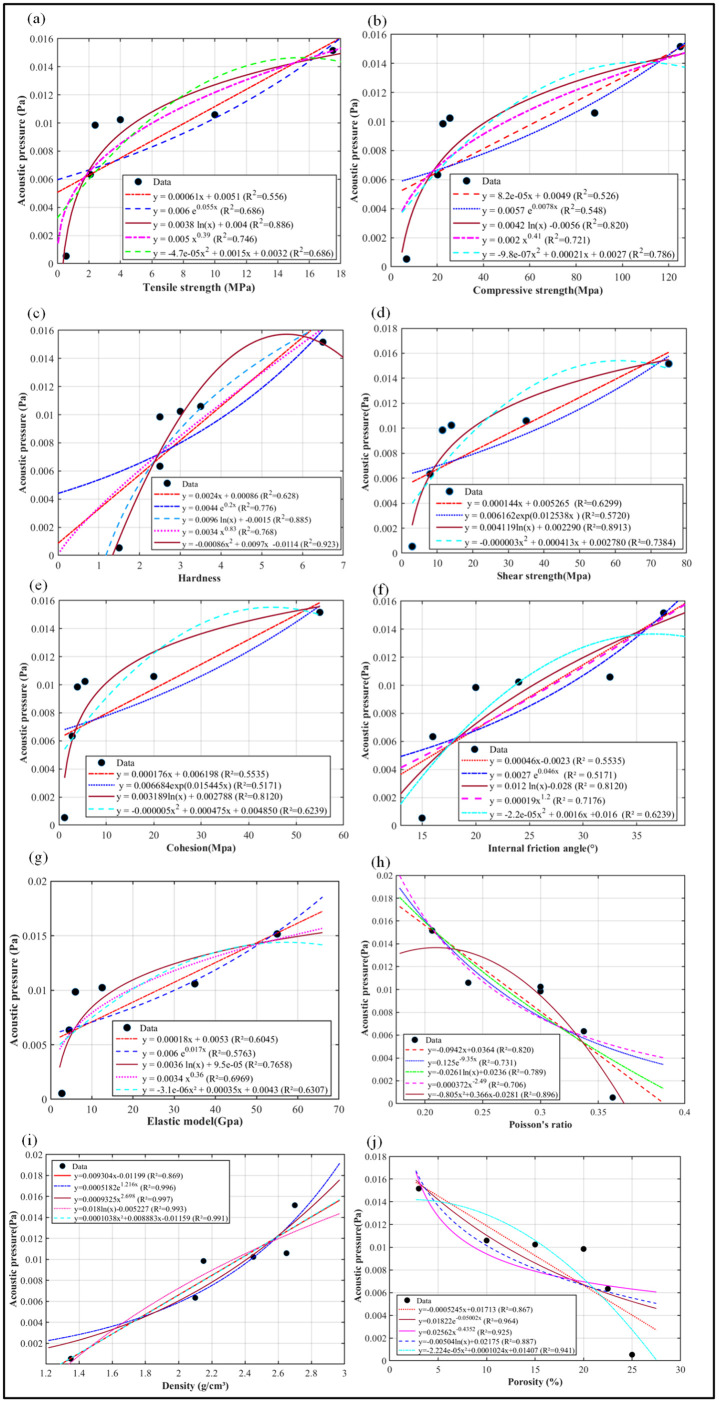
The acoustic pressure–physical parameter fitting model. (a) The acoustic pressure-tensile strength fitting model. (b)The acoustic pressure-compressive strength fitting model. (c)The acoustic pressure-hardness fitting model. (d)The acoustic pressure-shear strength fitting model. (e)The acoustic pressure-cohesion fitting model. (f)The acoustic pressure-internal friction angle fitting model. (g)The acoustic pressure-elastic modulus fitting model. (h)The acoustic pressure-Poisson ratio fitting model. (i)The acoustic pressure-density fitting model. (j)The acoustic pressure-porosity fitting model.

In this study, the mean of the denoised acoustic pressure was employed as the characteristic response value for each rock specimen. Using the least-squares method, quantitative models were fitted between this mean acoustic pressure and ten key physics parameters: tensile strength, compressive strength, hardness, shear strength, cohesion, internal friction angle, elastic modulus, Poisson’s ratio, density, and porosity, as illustrated in Fig 16.

Based on the fitting models presented in Fig 16, the model with the highest coefficient of determination (R²) was selected as the optimal representation for the relationship between acoustic pressure and each rock physical parameter. Specifically, logarithmic models best described the relationships between acoustic pressure and tensile strength (R² = 0.8860), compressive strength (R² = 0.8200), shear strength (R² = 0.8913), cohesion (R² = 0.8120), internal friction angle (R² = 0.8120), and elastic modulus (R² = 0.7658). Second-order polynomial models provided the best fit for hardness (R² = 0.9230) and Poisson’s ratio (R² = 0.8960). A power model showed the strongest correlation with density (R² = 0.9970), while an exponential model best fit the relationship with porosity (R² = 0.9640). These optimal fitting models are summarized in Table 7.

**Table 7:** Optimal fitting model of acoustic pressure-rock physics parameters.

Acoustic pressure- rock physical parameter	Fitting curves	R^2^
The acoustic pressure(P, Pa)-tensile strength(TS, MPa)	P=0.0038lnTS+0.0040	0.8860
The acoustic pressure(P, Pa)-compressive strength(CS, MPa)	P=0.0042lnCS−0.0056	0.8200
The acoustic pressure(P, Pa)-hardness(HM)	$P=-0.00086(HM{)}^{2}+0.0097HM-0.0114$	0.9230
The acoustic pressure(P, Pa)-shear strength(τ, MPa)	P=0.004119lnτ+0.002290	0.8913
The acoustic pressure(P, Pa)-cohesion(c, MPa)	P=0.003189lnc+0.002788	0.8120
The acoustic pressure(P, Pa)-internal friction angle(φ, ^0^)	P=0.012lnφ−0.028	0.8120
The acoustic pressure(P, Pa)-elastic modulus(E, GPa)	P=0.0036lnE+0.000095	0.7658
The acoustic pressure(P, Pa)-Poisson ratio(ν)	$P=-0.805\nu^{2}+0.366\nu -0.0281$	0.8960
The acoustic pressure(P, Pa)-density(ρ, g/cm^3^)	$P=0.0009325\rho^{2.698}$	0.9970
The acoustic pressure(P, Pa)-porosity(κ, %)	$P=0.01822e^{-0.05002\kappa }$	0.9640

### 3.4 Lithology identification analysis

Using the automatic lithology identification algorithm proposed in this study, an analysis was conducted on both homogeneous and layered drilling scenarios. This analysis aimed to validate the feasibility of the algorithm and evaluate the impact of transition zones in layered formations on its identification performance.

#### 3.4.1 Homogeneous drilling identification analysis.

**Fig 17:**
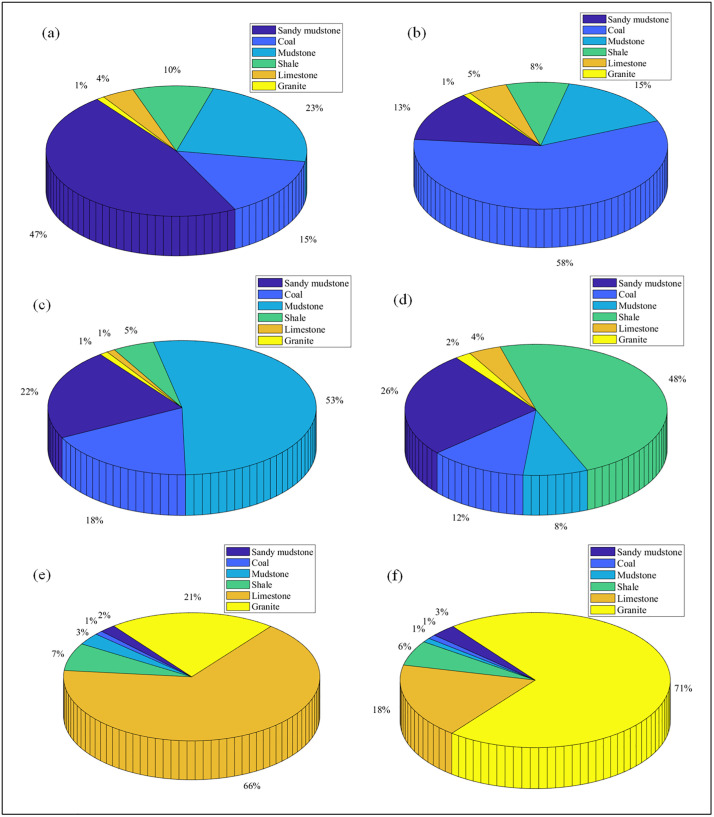
Comparison of lithology identification while drilling for homogeneous drilling. (a) Identification of sandy mudstone. (b) Identification of coal. (c) Identification of mudstone. (d) Identification of shale. (e) Identification of limestone. (f) Identification of granite.

The identification results for homogeneous drilling are shown in Table 8 and Fig 17. The recognition accuracies for sandy mudstone, coal, mudstone, shale, limestone, and granite were 47%, 58%, 53%, 48%, 66%, and 71%, respectively. In all cases, the correct lithology received the highest score, confirming the algorithm’s feasibility. Misidentifications occurred, primarily between rocks with similar physical properties. For example, sandy mudstone was misidentified as mudstone 23% of the time, which is attributable to the overlapping physical parameter ranges of these two rock types (see Table 4). However, despite these misclassifications, the correct lithology was always the most frequently identified class.

**Table 8:** Results of lithology identification for homogeneous drilling.

Lithology identification percentage (%)	Percentage of sandy mudstone identification (%)	Percentage of coal identification (%)	Percentage of mudstone identification (%)	Percentage of shale identification (%)	Percentage of limestone identification (%)	Percentage of granite identification (%)
Rock type						
Sandy mudstone	47	15	23	10	4	1
Coal	13	58	15	8	5	1
Mudstone	22	18	53	5	1	1
Shale	26	12	8	48	4	2
Limestone	2	1	3	7	66	21
Granite	3	1	1	6	18	71

#### 3.4.2 Layered drilling identification analysis.

**Fig 18:**
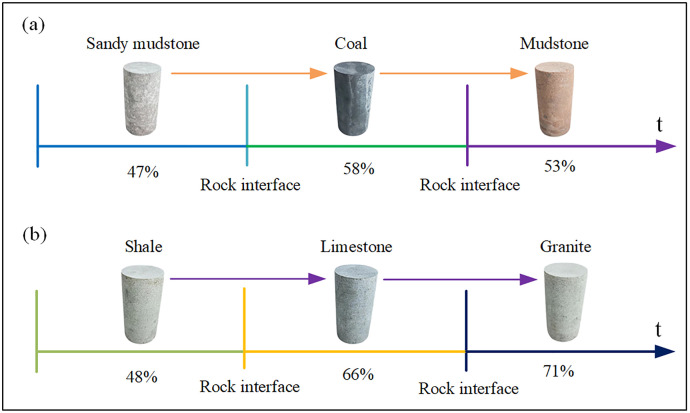
Lithology identification of through-layer boreholes while drilling. (a)Sequence of layered drilling: sandy mudstone-coal-mudstone. (b) Sequence of layered drilling: shale-limestone-granite.

As shown in Fig 18, in the lithology identification of cross-layer boreholes while drilling, the lithology identification percentages of sandy mudstone, coal, mudstone, shale, limestone, and granite are 47%, 58%, 53%, 48%, 66%, and 71% respectively, which are consistent with the lithology identification results of single-layer boreholes while drilling. The consistency between the lithology identification results of single-layer boreholes and cross-layer boreholes is attributed to the fact that although there are three cases of calculated physical parameter values of rock samples at the transition zones of cross-layer boreholes (as analyzed in Section 2.3.2), these cases only occur at the transition zones of cross-layer boreholes and only appear once (such as Identification Zone 3 and Identification Zone 6 in Fig 11(b)). Therefore, when counting the percentage scores of rock samples, the existence of transition zones in cross-layer boreholes does not affect the lithology identification of the automatic lithology identification algorithm while drilling.

## 4. Discussion

### 4.1 Comparison with previous work

#### 4.1.1 Comparison of experimental design.

This study addressed key limitations of previous experimental research:

(1) Rock Specimen Preparation: Previous studies often used artificially mixed materials and simple stacking for layered specimens [21,22], which deviate from in-situ rock. In contrast, this study used raw cores from an actual mine and prepared composite specimens with an integral sealing method, ensuring our samples more closely resembled real geological formations.
(2) Drilling Equipment Scale: Prior work frequently employed small-scale drilling rigs [23], creating a significant mismatch with field engineering. Our use of full-scale, industry-standard equipment ensured that the experimental conditions and collected data were more representative of actual drilling operations.
(3) Drilling Fluid System: Many lab studies have ignored the critical role of drilling fluid [21]. Our inclusion of a full drilling fluid circulation system allowed for a systematic evaluation of its effects on bit cooling, cuttings transport, and signal generation.

Overall, these enhancements in specimen preparation, equipment selection, and system configuration resulted in a laboratory setup that more faithfully simulates field conditions, yielding data with higher engineering relevance.

#### 4.1.2 Comparison of identification method.

Current methods for lithology identification predominantly rely on machine learning models like neural networks [24,25], deep learning [26,38], and support vector machines [39]. These approaches are data-hungry, requiring large datasets for training, which makes them costly. The method proposed in this study, however, uses acoustic pressure as a response feature and rock physics parameters as the identification basis. By leveraging the mapping relationship between them, our algorithm avoids the need for extensive training data, offering a more cost-effective solution.

### 4.2 Contributions

The main contributions of this research are four-fold:

(1) It established acoustic pressure as a viable and effective response feature for lithology identification while drilling.
(2) It constructed robust fitting models between acoustic pressure and rock physics parameters, revealing their underlying physical mechanisms.
(3) It designed a full-scale laboratory drilling system that accurately simulates field conditions.
(4) It developed a novel automatic lithology identification algorithm based on acoustic pressure-physical parameter mapping that is not dependent on large datasets.

### 4.3 Significance

This research focuses on a series of innovations in the automatic identification of lithology while drilling, which have theoretical value and practical significance for promoting the development of intelligent drilling in coal mines.

At the theoretical level, the introduction of acoustic pressure as a response feature broadens the range of physical quantities used for lithology identification, offering a new analytical perspective. The establishment of quantitative models and the elucidation of their physical mechanisms deepen the fundamental understanding of wave-rock interactions, providing a theoretical reference for smart drilling technologies.

In practical applications, the designed laboratory system provides a reliable platform for developing and validating identification algorithms by more accurately simulating field conditions. The proposed algorithm reduces the reliance on massive datasets, lowering identification costs and providing a practical solution for real-time lithology identification in the complex geological environments of coal mines. These outcomes are highly significant for the realization of intelligent drilling in the coal industry.

### 4.4 Future work

Future research will focus on expanding the variety of rock types tested to further refine the fitting models, then developing a comprehensive and robust automatic lithology identification system based on the acoustic pressure-physical parameter mapping principle. The research will be carried out specifically from the following two aspects:

(1) Test the algorithm at multiple construction drilling sites underground in coal mines to verify its engineering applicability.
(2) Study the response law of sound pressure and rock physical parameters under the coupling effect of underground environmental factors such as temperature and pressure, and improve the environmental adaptability of the model.

## 5. Conclusions

This study proposed and validated a method for automatic lithology identification while drilling based on the mapping between acoustic pressure and rock physics parameters. Through time-domain, frequency-domain, and denoising analyses of acoustic pressure data, followed by model fitting and algorithm development, the following conclusions are drawn:

(1) Acoustic pressure can serve as an effective response characteristic for lithology identification while drilling. Different rock samples exhibit significant differences in their acoustic pressure time-domain and spectral characteristics, and the greater the difference in the properties of the rock samples, the more obvious the difference in their acoustic pressure intensity characteristics. Specifically, for rock samples with large property differences (such as coal and granite), their acoustic pressure time-domain waveforms show more intense fluctuations and more prominent differences in intensity. In the frequency domain, the acoustic pressure peaks at 350 Hz differ significantly among different rock samples, which can be used as a key indicator for lithology identification. However, the peaks at 225 Hz and 450 Hz are the natural resonance frequencies of the drilling system and have no correlation with lithology.
(2) A strong correlation exists between acoustic pressure and rock physics parameters. Specifically, logarithmic models well described the relationships with tensile strength (R² = 0.8860), compressive strength (R² = 0.8200), shear strength (R² = 0.8913), cohesion (R² = 0.8120), internal friction angle (R² = 0.8120), and elastic modulus (R² = 0.7658). Second-order polynomial models were found for hardness (R² = 0.9230) and Poisson’s ratio (R² = 0.8960). Furthermore, a power-law relationship was identified for density (R² = 0.9970), and an exponential relationship for porosity (R² = 0.9640).
(3) The proposed automatic identification algorithm is feasible and effective. It achieved recognition accuracies of 47% (sandy mudstone), 58% (coal), 53% (mudstone), 48% (shale), 66% (limestone), and 71% (granite). In all tests, the correct lithology received the highest identification score.
(4) The automatic lithology identification algorithm while drilling exhibits stability in lithology recognition. In the lithology identification of cross-layer boreholes while drilling for sandy mudstone, coal, mudstone, shale, limestone, and granite, the recognition percentages are 47%, 58%, 53%, 48%, 66%, and 71% respectively, which are consistent with the lithology identification results of single-layer boreholes while drilling. At the transition zones of cross-layer boreholes, although there are three cases of calculated physical parameter values of rock samples, these cases only occur at the transition zones of cross-layer boreholes and appear only once. When counting the rock sample recognition percentages, the existence of transition zones does not affect the lithology recognition of the automatic lithology identification algorithm while drilling.

## Supporting information

S1 Data S_Data. (ZIP)
